# Integration of Engineered “Spark-Cell” Spheroids for Optical Pacing of Cardiac Tissue

**DOI:** 10.3389/fbioe.2021.658594

**Published:** 2021-06-18

**Authors:** Christianne J. Chua, Julie L. Han, Weizhen Li, Wei Liu, Emilia Entcheva

**Affiliations:** Cardiac Optogenetics & Optical Imaging Lab, Department of Biomedical Engineering, The George Washington University, Washington, DC, United States

**Keywords:** spheroids, optogenetics, induced pluripotent stem-cell-derived cardiomyocytes, channelrhodopsin-2, all-optical electrophysiology, optical mapping, pacing, cryopreservation

## Abstract

Optogenetic methods for pacing of cardiac tissue can be realized by direct genetic modification of the cardiomyocytes to express light-sensitive actuators, such as channelrhodopsin-2, ChR2, or by introduction of light-sensitized non-myocytes that couple to the cardiac cells and yield responsiveness to optical pacing. In this study, we engineer three-dimensional “spark cells” spheroids, composed of ChR2-expressing human embryonic kidney cells (from 100 to 100,000 cells per spheroid), and characterize their morphology as function of cell density and time. These “spark-cell” spheroids are then deployed to demonstrate site-specific optical pacing of human stem-cell-derived cardiomyocytes (hiPSC-CMs) in 96-well format using non-localized light application and all-optical electrophysiology with voltage and calcium small-molecule dyes or genetically encoded sensors. We show that the spheroids can be handled using liquid pipetting and can confer optical responsiveness of cardiac tissue earlier than direct viral or liposomal genetic modification of the cardiomyocytes, with 24% providing reliable stimulation of the iPSC-CMs within 6 h and >80% within 24 h. Moreover, our data show that the spheroids can be frozen in liquid nitrogen for long-term storage and transportation, after which they can be deployed as a reagent on site for optical cardiac pacing. In all cases, optical stimulation was achieved at relatively low light levels (<0.15 mW/mm^2^) when 5 ms or longer pulses were used. Our results demonstrate a scalable, cost-effective method with a cryopreservable reagent to achieve contactless optical stimulation of cardiac cell constructs without genetically modifying the myocytes, that can be integrated in a robotics-amenable workflow for high-throughput drug testing.

## Introduction

Traditionally, cardiac tissue is stimulated using electrodes delivering electrical pulses. Such electrodes require physical contact and cannot easily be deployed for multisite stimulation. Advances in optogenetics (Nagel et al., 2003; Boyden et al., 2005; Deisseroth et al., 2006; Zhang et al., 2006) present an alternative – pacing tissue using light. Optogenetic modification *via* infection or transfection to introduce light-sensitive ion channels or opsins, such as channelrhodopsin-2 (ChR2), in cardiomyocytes allows pacing by light that offers certain benefits over electrical stimulation (Williams and Entcheva, 2015). Optogenetic rhythm control has been deployed at the whole heart in a variety of studies (Arrenberg et al., 2010; Bruegmann et al., 2010, 2016; Zaglia et al., 2015; Crocini et al., 2016; Nyns et al., 2019). When combined with human induced pluripotent stem-cell-derived cardiomyocytes, human stem-cell-derived cardiomyocytes (hiPSC-CMs), *in vitro*, the contactless and scalable light-based optogenetic approaches hold promise to aid high-throughput (HT) capabilities for functional drug cardiotoxicity testing or other aspects of drug development (Lapp et al., 2017; Rehnelt et al., 2017; Zhang and Cohen, 2017; Entcheva and Kay, 2021). All-optical electrophysiology is poised to accelerate and streamline drug development (Hochbaum et al., 2014; Entcheva and Bub, 2016; Klimas et al., 2016, 2020).

Direct transduction methods generally require several days for the cardiomyocytes to express the opsin and to become responsive to the pacing rhythm of blue-light pulses (Ambrosi and Entcheva, 2014; Ambrosi et al., 2015, 2019; Lapp et al., 2017; Rehnelt et al., 2017). When done on site, they require special institutional protocols for handling recombinant DNA. On the other hand, it has been demonstrated that dedicated ChR2-expressing non-myocytes can be used as a driver to pace nontransduced myocytes by the so called “tandem-cell-unit” (TCU) approach (Jia et al., 2011; Ambrosi et al., 2015; Klimas et al., 2016; Boyle et al., 2021; Kostecki et al., 2021), Figure 1A, as shown earlier also for non-myocytes expressing HCN2 in tandem with cardiomyocytes (Valiunas et al., 2009). Some of these studies highlighted a benefit of reducing the energy needed for pacing by spatially aggregating the light-responsive cells by cell patterning (Ambrosi et al., 2015). This approach can simplify the requirements for using optogenetic pacing (no genetic modification will be required on site) and may shorten the time needed to achieve light responsiveness for HT drug testing applications.

**FIGURE 1:**
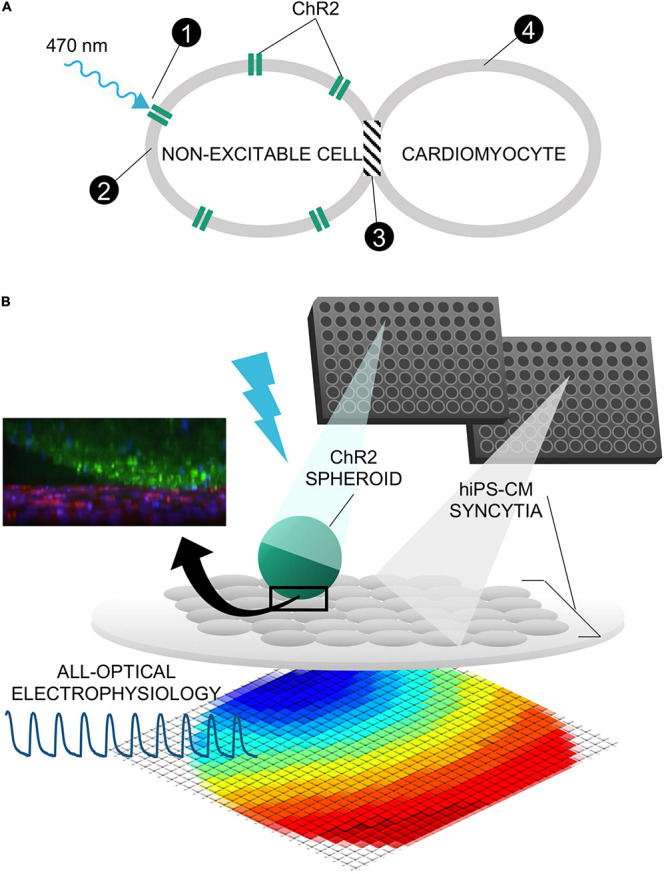
Tandem-cell-unit approach using three-dimensional spark cell spheroids. **(A)** Illustrated is the tandem-cell-unit (TCU) approach. (1) Blue light (470 nm) induces conformational change in ChR2 expressed in a non-excitable cell resulting in (2) inward current and an elevated membrane potential in the non-excitable cell. Because there exists (3) some form of coupling between the non-excitable and cardiac cells, (4) the system evokes an action potential and subsequent contraction in the cardiomyocytes. **(B)** Shown is the deployment of TCU using a spheroid structure and cardiac tissue – a schematic representation of a ChR2-expressing three-dimensional cell spheroid on top of a two-dimensional layer of hiPS-CM tissue. Confocal microscopy captured the relative positioning (ChR2-HEKs labeled green and hiPSC-CMs labeled red) as shown in the inset.

In this study, we demonstrate the manufacturing of three-dimensional constructs (spheroids) of ChR2-expressing “spark-cells” and characterize their properties, cryopreservation, integration, and ability to stimulate syncytia of hiPSC-CMs by the TCU strategy. The motivation for this study was twofold: (1) develop a modular system for contactless pacing that is scalable and amenable to robotic handling; and (2) avoid any genetic manipulation of the cardiomyocytes and demonstrate faster timecourse of “spark-cell” spheroid-mediated conferment of optical pacing compared to direct transduction techniques. Our intent for these “spark-cell” spheroids is to be ultimately used as a “reagent,” fitting into a manufacturing workflow with robotic handling, long-term storage, transportation, and reliable deployment in HT drug testing applications.

## Materials and Methods

### ChR2-HEK Spheroid Assembly

**FIGURE 2:**
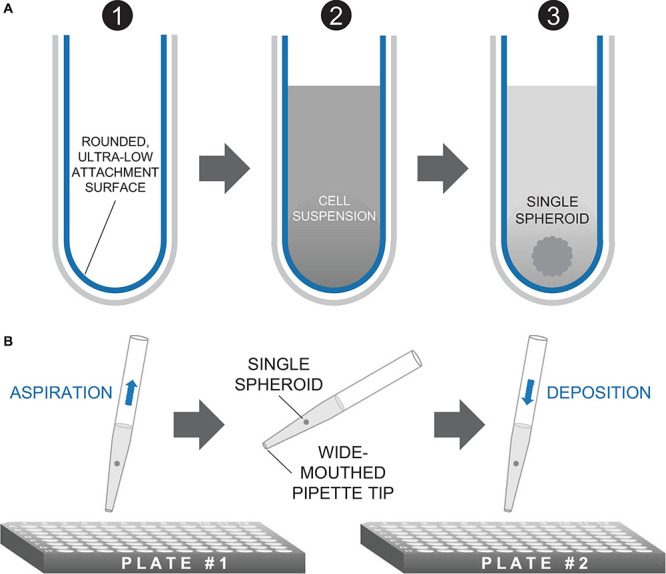
Method of spheroid formation. **(A)** Microplates featuring rounded, ultra-low attachment surfaces facilitate the formation of a three-dimensional spheroid of cells from a homogeneous cell suspension. **(B)** Spheroids are transferred in medium *via* standard micropipettors fitted with wide-mouthed tips.

Previously, we have developed an immortal 293T-HEK cell line expressing ChR2 with a YFP tag (Jia et al., 2011) using Addgene construct pcDNA3.1/hChR2(H134R)-EYFP, generously deposited by Karl Deisseroth. ChR2-HEKs were thawed, plated, and expanded in standard T-75 flasks in a Dulbecco’s Modified Eagle Medium (DMEM) supplied with 10% fetal bovine serum (FBS) and 1% penicillin-streptomycin. ChR2-HEKs were then dissociated from the T-75 tissue culture flasks by 0.05% trypsin in Hanks’ Balanced Salt Solution (HBSS) following a phosphate-buffered solution (PBS) rinse. After the resulting suspension underwent centrifugation, cells were resuspended in a volume of DMEM appropriate to yield spheroids of desired seeding density per well of a 96-well microplate (e.g., 10^2^ to 10^5^ cells/200 μL) or 384-well microplate (e.g., 5 × 10^2^ to 5 × 10^3^ cells/80 μL). Spheroids were cultured in Corning^®^ spheroid microplates in both 96-well (Catalog #CLS4920, Millipore Sigma) and 384-well (Catalog #CLS3830, Millipore Sigma) formats. These plates are uniquely designed with rounded, ultra-low-attachment surfaces to prevent cell adhesion while promoting self-assembly of cells into three-dimensional spheroids as shown in Figure 2A. Cell culture medium (DMEM + 10% FBS + 1% penicillin-streptomycin) was replaced every other day using a 50/50 approach (replace 50% of the medium) to minimize spheroid disturbance. Over 600 spheroids of different sizes were grown in different experimental rounds to optimize conditions and ultimately to produce data reported here. Growth of the spheroids was observed longitudinally *via* microscopy for as long as they remained viable.

### Characterization of Spheroids

**FIGURE 3:**
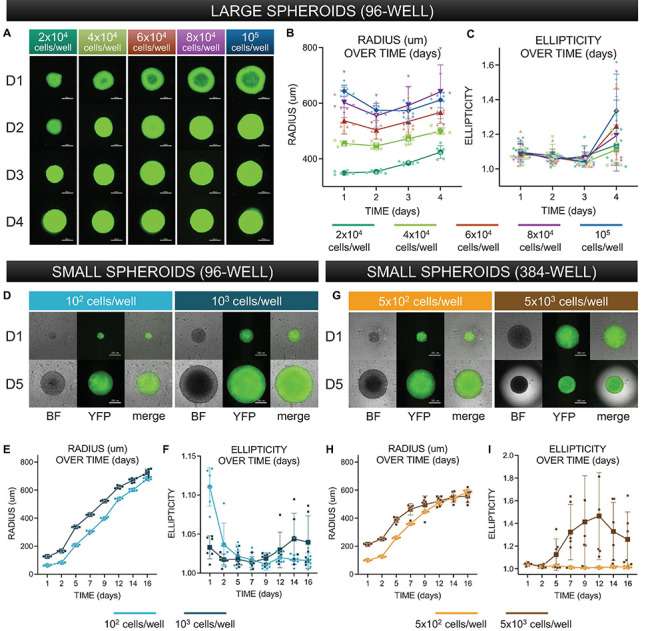
Morphological characterization of three-dimensional spheroids as function of cell density. **(A)** Assembled “large” spheroid constructs initially seeded at (G1) 2 × 10^4^ cells/well, (G2) 4 × 10^4^ cells/well, (G3) 6 × 10^4^ cells/well, (G4) 8 × 10^4^ cells/well, and (G5) 10 × 10^4^ cells/well. Both brightfield images and fluorescence images (to confirm expression of ChR2-eYFP) were taken every 24 h over 4 days. Scale bar is 0.5 mm. **(B)** Standard imaging tools were used to contour a five-point ellipse to the outline of the imaged spheroid. Axes were then used to calculate average radius and ellipticity for each spheroid over 4 days. **(C)** Radius [(major axis + minor axis)/2] as a function of time. **(D)** Shape (major axis/minor axis) as a function of time. For **(C–D)**, the sample size is 30 spheroids, *n* = 6 per density and per time point. **(D)** Assembled “small” spheroids with initial seeding of 10^2^ and 10^3^ cells per well in a 96-well format – brightfield and eYFP fluorescence images shown at days 1 and 5. Scale bar is 0.2 mm. **(E,F)** Radius and ellipticity over 16 days. The sample size was 12 spheroids, *n* = 6 per density and per time point. **(G)** Assembled “small” spheroids with initial seeding of 5 × 10^2^ and 5 × 10^3^ cells per well in a 384-well format – brightfield and eYFP fluorescence images shown at days 1 and 5. **(H,I)** Radius and ellipticity over 16 days. The sample size was 12 spheroids, *n* = 6 per density and per time point. Scale bar is 0.2 mm for day 1 and 0.5 for day 5. In panels **(B,C,E,F,H,I)**, all data points are overlaid in addition to the mean and standard deviation. See Supplementary Figures 1–4 as a function of time. For for complete large spheroid, 96-well format dataset. See Supplementary Figure 7 for complete small spheroid, 96-well and 384-well format datasets. Please see text and Supplementary Figure 8 for details on statistics.

Beginning 24 h after seeding, each spheroid was imaged on an inverted Nikon Ti2 microscope, using a 4× objective and an Andor 512 × 512 EMCCD camera under brightfield and fluorescence using an YFP filter (for visualization of ChR2-eYFP tag) in 24-h increments, as shown in Figure 3A (see Supplementary Figures 1–4 for complete dataset with brightfield and fluorescent images). For smaller spheroids such as those shown in Figures 3D,G, spheroids were imaged in the same way at days 1, 2, 5, 7, 9, 12, 14, and 16.

Morphological analysis (manual approach): Image analysis tools in NIS Elements (Nikon microscopes image acquisition software) allowed us to acquire spatial measurements for major and minor axes using a five-point ellipse estimation on spheroid images. Size and shape are defined by Eqs 1 and 2, respectively.

(1) $$radius=\frac{majoraxis+minoraxis}{2}$$

(2) $$ellipticity=\frac{majoraxis}{minoraxis}$$

Morphological analysis (automated approach): Due to the rapid growth of the spheroid image datasets, we eventually opted for an automated approach for quantifying spheroid size and shape. This was achieved in MATLAB *via* threshold binarization and automated morphological quantification of multiple image features. We extracted features analogous to those used in the manual approach, major and minor axes, and computed radius and ellipticity in the same way detailed above. All morphological plots shown in Figure 3 result from measurements obtained by this automated computational approach, though tables in Supplementary Figures 1–4 show data from manual measurements. The automated image analysis of spheroid shape was validated by the manual approach.

To evaluate spheroid viability, we utilized DNA-binding propidium iodide (PI) (Catalog #P3566 Thermo Fisher Scientific). PI is impermeable to cells and thus commonly used to identify dead cells.

For PI quantification experiments, WT-293T and ChR2-293T spheroids were grown in 2 μg/mL PI in DMEM at 2 × 10^4^ cells/well. Following imaging every 24 h, medium was replenished using our previously described 50:50 method using diluted PI instead of pure DMEM.

Imaging was performed under brightfield and fluorescence to evaluate PI saturation and ChR2-eYFP expression. After normalizing acquisition parameters, we calculated fraction of PI to spheroid area. The PI area was used as a numerator and the spheroid area from the brightfield was used as a denominator. To overcome challenges with finding an appropriate threshold to identify the silhouette of the spheroid, for the brightfield images, we used an auto-detect ROI feature in NIS Elements to identify the general outline of the spheroid and register the binarization to an exported image. This direct binarization and automated ROI method for image processing are depicted in Supplementary Figure 6. Using this adjusted parameter, we calculated the fraction of PI for each image in Supplementary Figure 5B (bottom). Images of control conditions (no PI stain) for both cell lines are also included in Supplementary Figure 5A.

### Culture of Stem-Cell-Derived Cardiomyocytes

Human induced pluripotent stem-cell-derived cardiomyocytes, hiPSC-CMs (Catalog #R1017, Fujifilm – Cellular Dynamics) were thawed and plated according to the supplied protocol. Prior to thawing, a 50 mg/mL solution of fibronectin diluted in sterile PBS was applied to all wells or plates which were then incubated overnight at 37°C. Maintenance medium was replaced every 48 h. For initial and integration experiments, the hiPSC-CM cells were plated in 96-well glass bottom plates at 50 × 10^3^ cells/well. For macroscopic experiments, hiPSC-CMs were seeded into 14 mm glass-bottom dishes at a seeding density of 270 × 10^3^ cells/well.

### Deposition of Spheroids onto Cardiac Syncytia

Channelrhodopsin-2-HEK spheroids at 2 × 10^4^ cells/well initial seeding density and cultured for 24 h were transferred *via* pipetting onto hiPS-CM syncytia, plated 5 days before, or 7 days prior, if transfection with an optogenetic sensor was applied. For cryopreservation experiments, spheroids with initial plating density of 10^2^ to 5 × 10^3^ cells/well were used for pacing after thawing. We utilized a wide-mouthed pipette tip with slow aspiration and deposition to minimize mechanical disturbance to the cell construct, as shown in Figure 2B.

### Immunocytochemistry

Immunocytochemistry was used to visualize the spheroid-syncytia constructs. The ChR2-expressing spheroids have an eYFP fluorescent reporter. For the hiPSC-CMs, we used the monoclonal anti-α-actinin antibody (Catalog #A7811, Millipore Sigma) to label cardiomyocyte sarcomeres and Hoechst (Catalog # H3570, Thermo Fisher Scientific) to label nuclei. After rinsing with PBS, cells were permeabilized in 0.2 Triton X-100 in 5% FBS and two-stage antibody labeling was applied to image the samples on an inverted microscope.

### Functional Experiments Using All-Optical Electrophysiology

**FIGURE 4:**
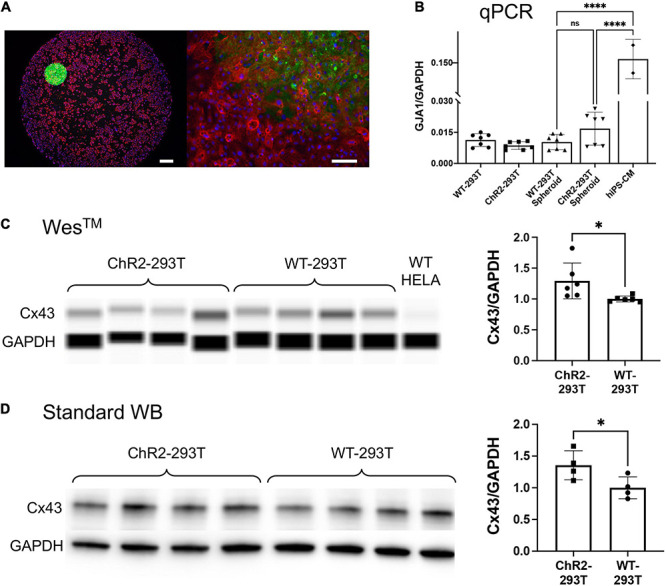
Spheroid deployment and coupling to human iPSC-CM syncytia. **(A)** Immunofluorescent image of a spheroid deposited onto a hiPSC-CM layer. Alpha-actinin (red) identifies the actin filaments in the sarcomeres of the myocytes; Hoechst (blue) labels the cell nuclei; eYFP (green) reporter identifies the expression of ChR2 which is localized in the HEK cell membrane. Left scalebar is set to 0.5 mm. Right scalebar is at higher magnification set to 0.1 mm. **(B)** Quantification of gap junction (GJA1) gene expression *via* qPCR (normalized by GAPDH). Shown are data from *n* = 7 independent samples of WT HEK and ChR2-HEK cells grown in monolayers and in spheroids, as well as *n* = 2 samples of hiPSC-CMs. **(C,D)** Western blot quantification (by capillary-based Wes^TM^ and by standard electrophoresis-based WB) of Cx43 gap junctional protein from WT HEK and ChR2-HEK cells, normalized by GAPDH protein. In the Wes^TM^ quantification, *n* = 6 per group, plus WT HeLA cells as a negative control; in the standard WB, *n* = 4 per group. Significant differences (*p* < 0.05) are indicated by a (*). ****p < 0.0001.

Functional experiments were conducted using all-optical electrophysiology using voltage or calcium small-molecule dyes or genetically encoded calcium sensors (Klimas et al., 2020; Li et al., 2020). Briefly, all functional experiments were performed in Tyrode’s solution (in mM): NaCl, 135; MgCl_2_, 1; KCl, 5.4; CaCl_2_, 1.8; NaH_2_PO_4_, 0.33; glucose, 5.1; and HEPES, 5 adjusted to pH 7.4. For voltage measurements, 24 h grown ChR2-HEK spheroids of 2 × 10^4^ cells per well were deposited onto hiPSC-CM syncytia in a 96-well format. After 12 h of integration, the samples were labeled with small-molecule near-infrared voltage-sensitive BeRST1 dye, courtesy of Evan W. Miller (Huang et al., 2015) at 1 μM concentration, as done in previous studies (Klimas et al., 2020). For measurements of intracellular calcium, we used either Rhod-4AM (AAT Bioquest, Sunnyvale, CA, United States) at 10 μM or one of two genetically encoded calcium indicators (GECIs)—R-GECO or its improved version, jRGECO—expressed in the hiPSC-CMs before the deposition of the spheroids. R-GECO [Addgene plasmid #45494 CMV-R-GECO-1.2, courtesy of Robert Campbell (Dana et al., 2016)] or jRGECO [Addgene plasmid #61563 pGP-CMV-NES-jRGECO1a, courtesy of Douglas Kim (Dana et al., 2016)] expression was done *via* transfection into the hiPSC-CMs with Lipofectamine 3000 at 400 ng per sample. All microscopic measurements were done on an inverted microscope Nikon Eclipse Ti2 at 20×. Blue light 470 nm *via* a digital micromirror device DMD (Polygon 4000, Mightex, Toronto, ON, Canada), 5–20 ms pulses at <0.15 mW/mm^2^ and using 0.5–2.0 Hz in order to trigger persistent excitation in the iPSC-CMs. Shorter pulses (down to 0.5 ms) were also used to construct strength-duration curves with a subset of spheroids, as shown in section “Results.” Excitation for voltage imaging with BeRST1 was at 660 nm and emitted light was collected using a long-pass filter at 700 nm, excitation for calcium imaging with Rhod-4AM, R-GECO and jRGECO was at 535 nm and emission was collected at 605 nm, in both cases with an iXon Ultra 897 EMCCD camera (Andor Technology Ltd., Belfast, United Kingdom), run at 200 fps. To avoid potential spectral overlap in the R-GECO and jRGECO measurements, we used patterned light, where the DMD stimulated region and the recorded region were physically different, as shown in Figure 4B. The patterns for optical stimulation (DMD targeted region) and optical sensing were selected manually for an initial sample in a way so that the former included the spheroid and the latter did not. These regions were kept the same as measurements were taken from different wells in the plate. Although in the current implementation, we did not automate the process [as we have done in earlier studies using ChR2-expressing hiPSC-CMs (Klimas et al., 2016, 2020)], it is possible to automate the spheroid positioning and then have these regions pre-programmed accordingly for HT screening.

### Probing Spheroid-Syncytia Coupling

After deposition of ChR2-HEK spheroids (2 × 10^4^ cells/well, 24 h) onto R-GECO-transfected hiPSC-CMs (10 days in culture including transfection with R-GECO), samples were transferred to an on-stage microscope incubator and probed for responsiveness to blue light (20 ms at 470 nm < 1 mW/mm^2^) pulsed at 0.5–2.0 Hz. Use of the incubator ensured temperature control at 37°C throughout the length of the 10–12 h experiment.

Functional tests with R-GECO as described in detail before were performed and repeated in 2-h increments over 10 h for a total of 6 time-probes. This procedure allowed us to determine the emergence of system responsiveness to optical stimulation by examining the number successfully stimulated samples over time.

### Macroscopic Optical Mapping

For imaging the macroscopic waves triggered by the spark-cell spheroids, we deposited the ChR2-293T spheroids (2 × 10^4^ cells/well, 24 h) onto hiPSC-CM samples plated in 14 mm glass-bottom dishes after 24 h of integration.

The widefield optical mapping of cardiac excitation wave was built around an MVX10 MacroView, Olympus, Japan system. We used a high-speed CMOS camera (Basler, Ahrensburg, Germany) to track wave dynamics. Either Rhod-4AM or R-GECO were used as calcium sensors for these measurements. Continuous fluorescence excitation light with an irradiance of 0.28 mW/mm^2^ was generated by a collimated green LED (M530L3, Thorlabs, United States), which obliquely illuminated the hiPSC-CMs dish from underneath. Pulsed optical pacing light with an irradiance of 0.35 mW/mm^2^ was generated by a weakly focused LED (M470L4, Thorlabs, United States), which was driven at 0.5 Hz (or 1 Hz) with a pulse duration of 10 ms. Microscopic and macroscopic recordings were processed using Matlab software for filtering and visualization (Bien et al., 2006; Klimas et al., 2016).

### Gene Expression Analysis by qPCR

Cells were plated on 96-well format. Two days post plating, cells were harvested for RNA extraction and mRNA levels were detected and quantified using Power SYBR^TM^ Green Cells-to-C_*T*_^TM^ Kit (Cat. 4402953, Invitrogen) according to the manufacturer’s protocol. qPCR analysis was performed on a QuantStudio 3 Real-Time PCR System (Thermo Fisher Scientific) with the QuantStudio Design and Analysis Software (Thermo Fisher Scientific).

Quantification of gap junctional gene expression of GJA1, encoding for Cx43, (primers: Fw_GGTGGTACTC AACAGCCTTATT; Rev_ACCAACATGCACCTCTCTTATC) was normalized to expression of housekeeping gene GAPDH (primers: Fw_GGAGCGAGATCCCTCCAAAAT; Rev_GGCTGTTGTCATACTTCTCATGG) using standard ΔΔCt method.

### Protein Quantification

Cells were lysed for total protein in 96-well format using the Qproteome Mammalian Protein Prep Kit (Cat. 37901, Qiagen). Protein lysates were loaded onto and analyzed using either traditional gel electrophoresis-based western blot or the Wes^TM^ (ProteinSimple) capillary-based system for protein quantification. The Wes^TM^ system allows for protein quantification in small samples in a semi-automated way; more details on both methods can be found in an earlier study with different antibodies (Li et al., 2020). Proteins of interest were probed using antibodies specific to Cx43 (ab11370, Abcam) and GAPDH (ab181602, Abcam). Changes of Cx43 protein levels were presented as normalized to GAPDH.

### Spheroid Cryopreservation for Long-Term Storage

Spheroids grown in ultra-low-adhesion microplates (96 well and 384 well) were optimized by size for purposes of freezing/thawing. We established that smaller spheroids (with <10^4^ cells at plating; initial diameter <0.3 mm) performed better when frozen and thawed. After optimization, we utilized freezing medium with the following composition (in %): DMSO:DMEM:FBS (10:30:60). After 24 h of plating in the ULA microplates (96 well or 384 well), spheroids were transferred to this freezing medium in cryovials. First, controlled cooling 1°C/min was applied overnight in a −80°C freezer. After that, the cryovials with spheroids were transferred to liquid nitrogen (−120°C) for long-term storage (7–10 days in this study).

Quick thawing (using a 37°C water bath) was applied and the spheroids were transferred to DMEM culture medium in 96-well ULA microplates. Propidium iodide (2 μg/ml) was added to monitor viability. Spheroids were recovered for 4 days and then administered to monolayers of human iPSC-CMs in 96 well format. Functional testing (using optogenetic sensor jRCEGO for intracellular calcium and blue pulses of light for pacing) was done within 18 h after depositing the spheroids.

### Statistics

All statistical analysis was performed in GraphPad Prism. Changes in spheroid morphology over time and initially seeding density was evaluated for significance *via* two-way ANOVA with post-hoc Tukey or Sidak. Differences in gene expression between assessed groups was tested for significance *via* one-way ANOVA with *post hoc* Tukey. Finally, for protein quantification data by Wes^TM^ and standard western blot, an unpaired *t*-test was used. In all statistics, *p* < 0.05 and normality of the data was tested *via* Shapiro–Wilk test.

## Results

### Manufacturing of “Spark-Cell” Spheroids

Our goal was to scale the TCU strategy up, Figure 1A, into easy-to-handle, configurable three-dimensional structures of ChR2-expressing cells for use in HT drug screening of cardiac tissue. Such “spark-cell” spheroids composed of optogenetically transformed live cells can be positioned atop hiPSC-CM syncytia, as shown in Figure 1B, to mediate optical pacing.

While various methods exist to form three-dimensional cell constructs, including three-dimensional bioprinting with hydrogels and photopolymerizable materials (McBeth et al., 2017; Ashammakhi et al., 2019), we opted for spheroid formation *via* self-aggregation, due to its simplicity. Indeed, it has been demonstrated that cells deposited in agarose molds can self-assemble into three-dimensional tissue spheroids (Napolitano et al., 2007; Mehesz et al., 2011). We fabricated our ChR2-HEK spheroids in commercially available 96-well or 384-well microplates with ultra-low attachment treatment, as in Figure 2A. The assembly is further facilitated by gravity. It is possible to use wide-mouth pipette tips to handle these spheroids, i.e., to carefully lift, transfer, and to deposit them on top of hiPSC-CM monolayers (grown in 96-well plates), as shown in Figure 2B.

Optimization of seeding cell density included considerations for easy handling (the spheroids had to be large enough) and avoiding the formation of a significant necrotic core (not too large). Initial seeding density between 10^4^ and 10^5^ cells/well yielded viable and functional spheroids recognizable by the human eye and therefore easy to handle. However, with optimization of spheroid handling, we were able to generate spheroids of smaller seeding density, on the order of 10^2^ to 10^3^ cells/well in both 96-well format as well as in 384-well format.

### Characterization of Spheroids

Larger spheroids, formed using initial seeding densities from 2 × 10^4^ to 10^5^ cells/well, in increments of 2 × 10^4^ cells/well were explored. To register how spheroids evolved structurally over time, brightfield images were captured every 24 h, along with images of eYFP to monitor the expression of ChR2-eYFP, Figure 3A. For morphological analysis of the spheroids, we applied threshold binarization and morphological operations on the images to obtain quantitative parameters that could be used to describe the observable evolution of size and shape (ellipticity), Figures 3B,C. Large spheroids at the studied seeding densities became more compact over the first 48 h, then grew in size due to cell proliferation. A two-way ANOVA revealed that spheroid size was significantly impacted (*p* < 0.05) by both time of culture and seeding density. For these larger spheroids, ellipticity was not significantly impacted by seeding density but only by time in culture (*p* < 0.05), where at day 4, all groups showed significant deviations from a perfect sphere (captured here by increase in ellipticity). A subset of the statistics (pairwise comparisons using *post hoc* Tukey correction) is shown in Supplementary Figure 8.

Characterization of smaller spheroids was undertaken later in the study, as we optimized spheroid handling procedures. We reasoned that a smaller seeding density would allow better consistency of shape and size over time, leading to a longer window of spheroid usability and higher viability for storage applications. Shown in Figure 3D are smaller-size spheroids at seeding density 10^2^ and 10^3^ cells/well in 96-well plates at days 1 and 5. In the corresponding 16-day morphological analysis shown in Figures 3E,F, we observed a steady increase in radius but relatively little deviation from a perfect sphere. A two-way ANOVA showed significant dependence of spheroid size both on seeding cell density and on time in culture (*p* < 0.05); ellipticity, similar to the larger spheroids, was only influenced on time in culture but not on seeding density. Overall, these spheroids showed much lower deviations from a sphere over more than 2 weeks in culture. We also manufactured spheroids in 384-well plates; samples at 5 × 10^2^ and 5 × 10^3^ cells/well are shown at days 1 and 5 in Figure 3G. In this format, spheroid radius initially grew and then reached a size limit, as shown in Figure 3H, likely due to the higher curvature of the well. Similar to the larger spheroids (>10^4^ cells/well), after several days in culture, the ellipticity of the 5 × 10^3^ cells/well group (Figure 3I) increased substantially. All groups of smaller spheroids (up to 10^3^ cells/well) did not demonstrate any stages of compression, but rather steadily grew in size while maintaining perfect spherical shape over 2 weeks in culture, Figures 3E,F. Applying a two-way ANOVA, we found that spheroid size and ellipticity were influenced significantly (*p* < 0.05) by seeding density and time in culture. Pairwise comparisons are shown in Supplementary Figure 8. Overall, for a known plating cell density, spheroid size was highly reproducible as shown by the tight distributions in Figure 3, and these smaller spheroids were more versatile as they remained intact and close to a sphere within over 2 weeks.

In the large spheroids, we also attempted to assess viability of the constructs by culturing in DMEM, containing PI at 2 μg/mL, as Zhao et al. have shown the safe long-term use of PI (Zhao et al., 2010). Every 24 h, cells were imaged and medium replaced with the same PI dilution. This characterization experiment generated the images for Supplementary Figure 5, in which WT 293T and ChR2-infected 293T spheroids were seeded at 2 × 10^4^ cells/well monitored over time. Control group data (spheroids in PI-negative culture medium) in Supplementary Figure 5A is shown alongside experimental group data (spheroids in PI-positive culture medium) in Supplementary Figure 5B. Binarization of the spheroids with PI images revealed a quantitative PI fraction for each cell line and day (*n* = 3 for each condition), summarized at the bottom of Supplementary Figure 6. Spheroids developed a necrotic core with time in culture as shown by the localization of PI in the center of the structures. However, these necrotic cores were surrounded by a wall of viable cells, therefore this did not affect their acute use to pace cardiomyocytes.

### Gap Junctions and the “Spark-Cell” Spheroids

Immunocytochemistry and confocal imaging helped visualize the coupling of the spheroids with cardiomyocyte layers (Figure 4A). Of interest was the coupling mechanism between the ChR2-spheroid and hiPSC-CM syncytia in this larger-scale TCU approach. We expected that gap junctional proteins, such as Cx43 (abundantly expressed in ventricular cardiomyocytes), play a role, even though the HEK cells are known to have minimal amounts of Cx43. We used small-sample qPCR and protein quantification assays to probe for GJA1/Cx43 expression levels, normalized by GAPDH transcript/protein, respectively. As expected, the lysed monolayers and spheroids of WT-HEK and ChR2-HEK cells had an order of magnitude lower GJA1 mRNA levels (normalized to GAPDH) compared to the human iPSC-CMs, *p* < 0.05 by one-way ANOVA and Tukey *post hoc* comparisons, Figure 4B. Yet, the spheroids had detectable, albeit low, levels of GJA1. Furthermore, the ChR2 expression seemed to slightly increase GJA1 mRNA (n.s.), *n* = 7 biological replicates per group (with 3 technical replicates per sample). At the protein level (Figures 4C,D), ChR2-HEK also showed some increase in normalized Cx43 protein levels compared to the WT HEK, as it has been seen earlier (Jia et al., 2011). Specifically, unpaired *t*-test in standard western blot results, showed that ChR2-HEK had 35% higher Cx43/GAPDH levels compared to WT-HEK (*p* = 0.048, *n* = 4 biological replicates per group); in Wes^TM^ runs, about 41% increase was seen (*p* = 0.035, *n* = 6 biological replicates per group). The presence of some Cx43 in the “spark-cell” spheroids suggests gap junctional contribution to their integration with the human iPSC-CMs, although other mechanisms could be at play as well.

### Demonstration of Functionality

To optimize the stability of the larger spheroids, we determined that the most impactful constraints were early time in culture and low initial seeding density, e.g., 2 × 10^4^ cells/well. Hence, functional testing experiments were performed at this cell density after 24 h upon depositing the spheroids onto 96-well monolayers of hiPSC-CMs (pre-cultured for 5 days post-thaw) as described in Figure 1B.

**FIGURE 5:**
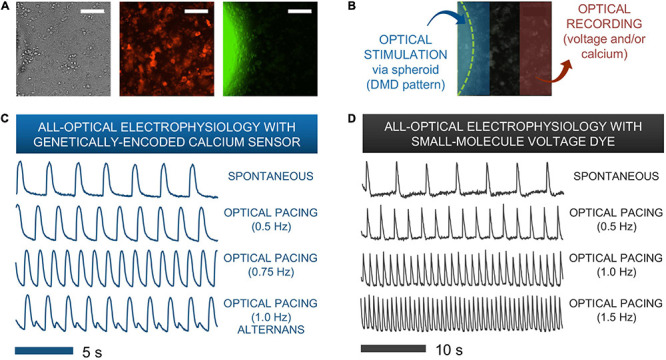
Functional responses from human iPSC-CMs upon spark-cell cluster pacing obtained by all-optical electrophysiology. **(A)** A typical field-of-view for microscopic optical stimulation experiments featuring brightfield and corresponding fluorescence images. The middle image was produced using a mCherry filter and demonstrates expression of optogenetic sensor for calcium (R-GECO) in the iPSC-CM monolayer. The rightmost image was produced with a YFP filter and shows a portion of the ChR2-eYFP spheroid. Scale bar is 0.1 mm. **(B)** For recordings, the field-of-view was separated into two non-overlapping areas – patterned optical stimulation including the spheroid and optical recording including only iPSC-CMs labeled with voltage or calcium indicators. **(C)** Calcium transients from R-GECO-expressing iPSC-CMs showing spontaneous activity and optical pacing (*via* the spheroid) at three different frequencies after 7 h of spheroid introduction. While a 1:1 stimulation-to-response is observed for optical pacing at 0.5 and 0.75 Hz, overdrive pacing at 1.0 Hz results in failure of iPS-CMs to fully restore calcium stores between stimulation events, hence calcium alternans are seen. **(D)** Optical pacing can also be confirmed *via* spectrally compatible small-molecule voltage dye. This sample shows a 1:1 relationship between stimulation and response for pacing frequencies up to 1.5 Hz after 12 h of spheroid integration.

Typical all-optical electrophysiology experiments (as in Figure 5) were done 12 h post spheroid deposition onto hiPSC-CMs grown in 96-well plates. For optical confirmation of pacing, we used two genetically-encoded calcium sensors (GECIs), R-GECO or jRGECO, expressed in the iPSC-CMs, or a near-infrared voltage-sensitive dye, BeRST1, both of which are spectrally compatible with the ChR2 actuator (Klimas et al., 2020; Entcheva and Kay, 2021). To further suppress potential cross-talk when using calcium recordings with R-GECO or jRGECO, we spatially patterned the light to confine the excitation near the spheroid and optically recorded from a nearby region, Figure 5B. Such restrictions were not necessary when using the red-shifted voltage-sensitive dye.

Figures 5C,D show traces of intracellular calcium (*via* R-GECO) and action potentials (*via* BeRST) from spontaneous and paced activity *via* “spark-cell” spheroids. Successful pacing was confirmed when samples followed 1:1 the selected frequencies over at least 10 consequitive beats. Incomplete capture sometimes resulted in alternans or 2:2 response (Bien et al., 2006), as shown in Figure 5C. Note that the maximum capture frequency is strongly dependent on temperature. In these experiments, performed at 25–30°C, typical maximum capture frequency was between 0.7 and 1.5 Hz. These responses are consistent with electrical pacing.

**FIGURE 6:**
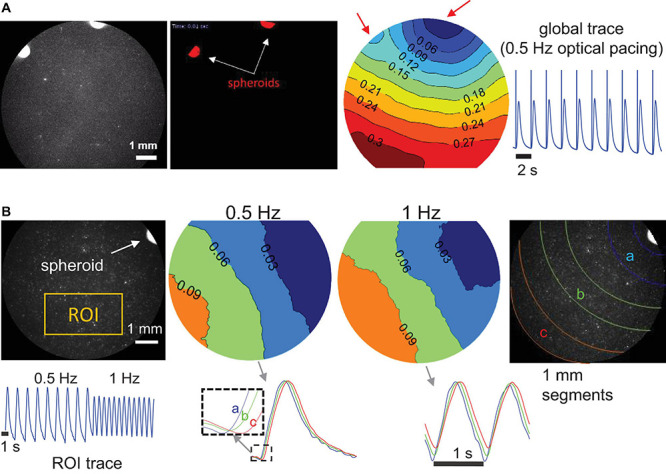
Macroscopic optical mapping of optically triggered spheroid-mediated calcium waves in human iPS-CMs. **(A)** Calcium waves (by Rhod-4) triggered by optical light pulses at 0.5 Hz through a pair of spheroids after 24 h integration. Activation map shows the origin of early activation (blue), red arrows indicate the locally triggered activity by the two spheroids; isochrones are 0.03 s apart. Global calcium transients and the superimposed optical pulses are shown on the right. **(B)** Calcium waves by optogenetic sensor R-GECO, triggered at 0.5 and at 1 Hz through a single spheroid (after 24 h integration). In the activation maps (isochrones 0.03 s apart) slight wave-slowing is visible at the higher pacing frequency. At the bottom, an ROI trace is shown for the uninterrupted pacing and traces from different segments away from the spheroid illustrate sequential activation (a, b, and c). Scale bar is 1 mm.

To confirm that the “spark-cell” spheroids are capable of pacing cells in larger cm-scale cardiac samples and to map the waves triggered by the spheroids, we also performed some macroscopic optical mapping experiments in 14 mm dishes, Figure 6. For these experiments, the spheroids were positioned on top of the cardiomyocytes 24 h before the imaging. Indeed, we were able to register spheroid-triggered excitation waves using either R-GECO or Rhod4-AM calcium sensor, as shown in Figure 6. Light was aimed at the spheroids but not focused on them. When more than one spheroid was used, as in Figure 6A, both initiated activity locally (red arrows) upon light trigger but a dominant pacemaker emerged, driving the cardiac syncytium on each beat.

### Timing of Integration

**FIGURE 7:**
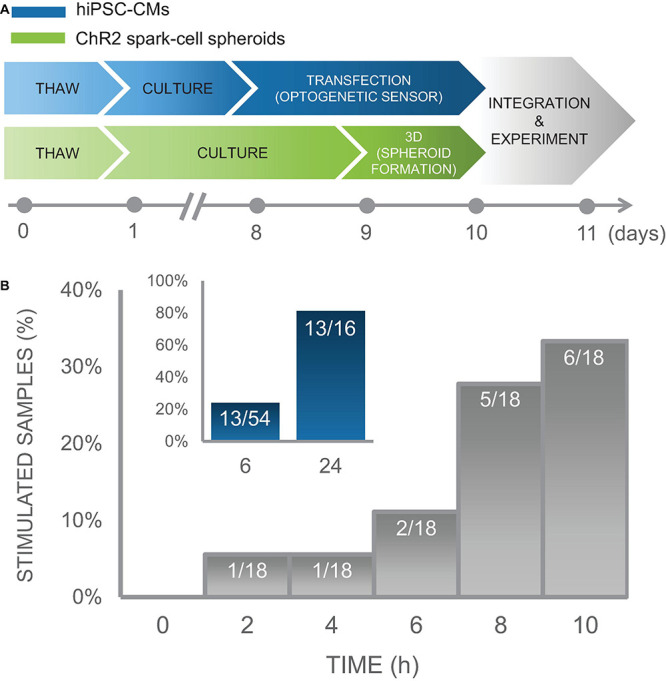
Time course of integration of spark-cell clusters with human iPS-CM monolayers for pacing capture. **(A)** Experimental preparation timeline including separate treatment of cell lines (hiPSC-CM in blue and ChR2 293Ts in green) before acute functional studies. **(B)** For an early integration study, 18 samples were tested and the percentage of those samples from which we observed full pacing capture was plotted every 2 h. We observed an increase in the number of optical pacing responders over 10 h. The inset (blue), represents the combined results over multiple cell cultures, summarized as a percent of samples that responded to optical pacing at 6 h (*n* = 54) and at 24 (*n* = 16) after spheroids were seeded onto hiPSC-CM syncytia.

After confirming spheroid functionality, we sought to probe the earliest emergence of TCU-based coupling in our system. To do so required the approximate timeline of experimental preparation shown in Figure 7A.

In an iPSC-CM culture of sample size of *n* = 18 biological samples, we performed measurements every 2 h over a 10 h window using on-stage incubator with temperature control to minimize potential mechanical disturbance from transferring samples to and from an external incubator and imaging system. In this culture, 1 out of 18 samples showed responsiveness to optogenetic pacing after only 2 h with an increasing number of samples coupling over the experiment. At the end of 10 h, 6 of the 18 samples showed responsiveness to optogenetic pacing. These results are shown in Figure 7B. Separate less involved experiments were conducted to capture integration efficiency at just 6 h and at 24 h post-spheroid introduction. Five of the 16 biological samples in this iPSC-CM set, were successfully paced optogenetically at 6 h; and 13 out of the 16 samples were responsive at 24 h. To increase sample size just at the 6 h time point, we tested the same responsiveness efficiency at 6 h post-spheroid introduction for additional 20 iPSC-CM cultures, for which 6 out of 20 samples demonstrated optical pacing at 6 h.

**FIGURE 8:**
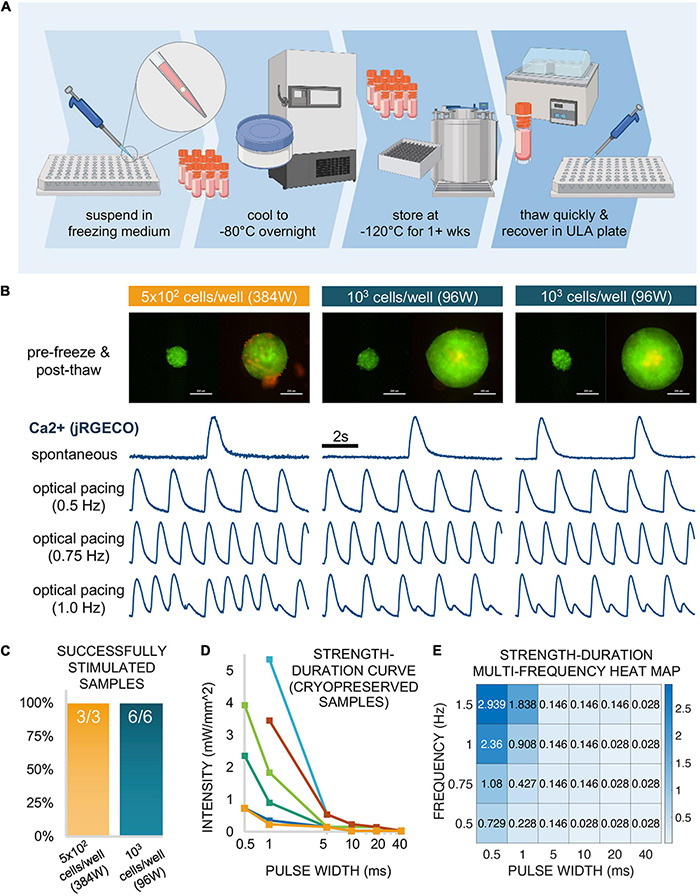
Functional responses *via* cryopreserved spark-cell clusters. **(A)** Schematic representation of the spheroid cryopreservation process, both freezing and thawing. **(B)** Sample spheroids (1 sample seeded in a 384-well spheroid plate at 5 × 10^2^ cells/well and 2 samples seeded in a 96-well spheroid plate at 10^3^ cells/well) from which functional data was obtained. Images (ChR2-eYFP and PI) represent spheroids 1 day before freezing and 6 days after thawing (or 1 day before functional measurements were performed). Scale bar is 0.2 mm. For each depicted spheroid are the corresponding calcium traces from jRGECO-expressing iPSC-CMs show spontaneous activity and responses to optical pacing by a spheroid at three frequencies 18 h after spheroids were applied. Stimulation at 0.5 and 0.75 Hz resulted in a 1:1 response ratio while varying patterns alternans were observed for these samples at 1.0 Hz. **(C)** Of the thawed spheroids that were introduced to iPSC-CMs, all (100%) of the samples demonstrated optical pacing. **(D)** A strength-duration (blue light intensity, pulse width) curve was constructed for 6/9 of the responsive cryopreserved samples at six discrete pulse widths: 40, 20, 10, 5, 1, and 0.5 ms. **(E)** For one of these six samples, a strength-duration heat map was constructed at multiple frequencies (0.5, 0.75, 1.0, and 1.5 Hz) where blue light intensity threshold (mW/mm^2^) for stimulation increases as pulse width decreases and/or frequency increases. Panel **(A)** was created using Biorender.

In total, we conducted experiments monitoring timing of integration over several iPSC-CM cultures for a total combinable dataset of *n* = 54 independent samples with various configurations of tested time points. These data demonstrate high efficiency of the “spark cell” spheroids to couple with cardiac syncytia, as 13 out of 16 (81%) iPSC-CM cultures (first subset only) yielded responsiveness after 24 h, and overall 13 out of the entire 54 (24%) iPSC-CM cultures (all three subsets) demonstrated functionality after only 6 h post-introduction of the spheroid to the cardiomyocytes. This summary data is shown in the inset of Figure 7B. Note that with optimization of the handling technique, we achieved 100% integration for the cryopreserved and thawed spheroids at 18 h post-deposition, see Figure 8. The early emergence of optogenetic pacing confirmed by these experiments confirms that our “spark cell” spheroid approach is able to yield functional optical pacing significantly earlier than the standard direct viral or liposomal transductions of the cardiomyocytes, which typically require 48 h.

### Spheroid Cryopreservation and Successful Deployment After Thawing

Of interest for this modular approach to contactless pacing was whether the spheroids could be used as “reagents” that can be stored, transported and deployed on demand. As long-term storage and transportation through cryopreservation has been a commonly used technique for cell suspensions, we sought to apply a similar approach to preserving these multi-cellular structures, as suggested for other applications (Kim et al., 2018).

Spheroids (on the order of 10^2^–10^3^) were frozen in a freezing medium consisting of 30% DMEM + 60% FBS + 10% DMSO in a controlled manner to −80°C overnight. The following day, samples were transferred to liquid nitrogen at −120°C for 1 week. Subsequently, they were successfully thawed and recovered in a 96-well spheroid microplate. This cryopreservation workflow is shown schematically in Figure 8A.

After the first day post-thaw, spheroids were labeled with PI diluted in medium for viability analysis, where medium was replaced every 48 h. Figure 8B shows three successfully recovered spheroids 1 day before freezing (left) and 6 days after thaw from liquid nitrogen (right). Of these three depicted samples, 1 spheroid had been seeded at 5 × 10^2^ cells/well in a 384-well spheroid plate while the other two spheroids had been seeded at 10^3^ cells/well in a 96-well spheroid plate. Though PI staining (red) revealed a small amount dead tissue on the surface of one of the samples, this did not affect the spheroids ability to couple with the cardiomyocytes. Indeed, all three depicted samples successfully elicited calcium transients as recorded my jRGECO in cardiomyocytes, with spontaneous as well as light-controlled pacing at different frequencies: 0.50 Hz (1:1 response), 0.75 Hz (1:1 response), and 1.0 Hz (alternans). In total, we successfully thawed and demonstrated spheroid functionality in nine cryopreserved samples, three spheroids at 5 × 10^2^ cells/well in a 384-well format and six spheroids at 10^3^ cells/well in a 96-well format as depicted in Figure 8C. Note that all nine out of nine tested samples achieved successful optical pacing within 18 h post-deposition.

From the six additional samples tested (not the 3 shown in Figure 8B), we constructed individual strength-duration curves (Figure 8D) to assess the blue light intensity and pulse width thresholds necessary for 1:1 coupling. As stated in the description for functional experiments, 20 ms pulses at very low light intensity (<0.1 mW/mm^2^) was sufficient for optical pacing, with higher intensities at shorter pulse widths, down to 0.5 ms. Note that the six samples needed quite different light levels when the pulses become very short. In a previous study, using fibroblasts and cardiomyocytes, we have shown experimentally and computationally that the irradiance needed to trigger optical pacing (at short pulses, in particular) can effectively be used to quantify coupling in such assemblies (Boyle et al., 2021).

For one of the six samples assessed in Figure 8D, we further explored the strength-duration threshold at multiple frequencies. The data for this particular sample is shown in Figure 8E as a heat map, where blue light intensity in mW/mm^2^ is a function of pulse widths (0.5, 1, 5, 10, 20, and 40 ms) and frequency (0.5, 0.75, 1.0, and 1.5 Hz). This data indicates >1 mW/mm^2^ intensity requirement for only four of the frequency-to-pulse width paired parameters, involving particularly short pulses of 0.5 and 1 ms.

Overall, our demonstration of functionality with spheroids that have undergone cryopreservation suggests that these “spark-cell” spheroids can be treated as transportable reagents that are storable and deployable on demand for on-site optical cardiac pacing.

## Discussion

High-throughput drug screening, and specifically cardiotoxicity testing, can benefit from optical actuation and sensing methods that offer contactless and scalable interrogation of cardiomyocytes. Optogenetic approaches play a key role in the development of HT all-optical electrophysiology. Some of the limitations associated with (opto)genetic modification of cells include potential interference with their innate functional responses and the time needed for the genetic modification to produce the protein of interest.

In this study, we sought to biomanufacture “spark-cell” spheroids, usable as a “reagent” that eventually can be stored, transported and deployed on site to confer optical pacing of cardiac tissue. Such “spark-cell” spheroids are amenable to automation and can be handled robotically. When deposited onto human iPSC-CM syncytia, they create a spatially localized pacing site. Our results show that this approach may offer faster integration for acute pacing experiments (as early as 2 h post-deposition in some cases) compared to direct genetic modification of the cardiomyocytes. The mechanism of optical pacing is based on the TCU approach (Valiunas et al., 2009; Jia et al., 2011) and likely involves the formation of close contact and gap-junction mediated ion currents between the spheroid and the responding cardiomyocyte syncytia.

Earlier studies have highlighted the utility of genetically modified non-myocytes in creating surrogate excitable systems for mechanistic studies of cardiac arrhythmias, in translational studies for optimizing genetically encoded voltage sensors etc. (Kirkton and Bursac, 2011, 2012; Park et al., 2013; McNamara et al., 2018). For the methodology outlined in the current study, additional optimizations can speed up the contact formation even further perhaps using magnetic assemblies (Kilgus et al., 2012; Lapp et al., 2017), microfluidic deposition or other augmentations, including the deployment of robotic manipulation. Furthermore, drawing on published work on spheroids made out of cardiac progenitor cells, cardiac fibroblasts or cardiac myocytes (Oltolina et al., 2015; Kim et al., 2018; Campbell et al., 2019; Grijalva et al., 2019), the proposed optogenetic approach can facilitate the assembly of modular designer tissues with space-patterned pacemakers in three-dimensional. It can find translational applications in the pharmaceutical industry for acute and chronic drug testing. The methodology can also be useful for aiding the long-term maturation of human iPSC-CMs *via* optical pacing for regenerative purposes.

## Data Availability Statement

All data used to construct the plots and run statistical analysis are included in the manuscript and shown in the Supplement; individual data points are plotted. Additional information can be obtained from the corresponding author upon reasonable request.

## Author Contributions

CC and EE conceived the project and designed the experiments. CC performed all spheroid fabrication and characterization experiments, the all-optical experiments for quantifying spheroid integration, cryopreservation optimization, and all data analysis, and wrote a first draft of the manuscript. JH cultured the iPSC-CMs and performed the qPCR and Wes experiments. WzL performed the immunostaining and helped with cell culture and Wes experiments. WL performed the macroscopic optical mapping and data analysis. EE provided reagents and oversaw the project. CC and EE edited the manuscript with input from all authors.

## Conflict of Interest

The authors declare that the research was conducted in the absence of any commercial or financial relationships that could be construed as a potential conflict of interest.
