# Supplementary material for: Integration of Engineered “Spark-Cell” Spheroids for Optical Pacing of Cardiac Tissue
Source: Front Bioeng Biotechnol. 2021 Jun 18;9:658594. doi: 10.3389/fbioe.2021.658594 (PMC8249938; doi:10.3389/fbioe.2021.658594)
Supplement: Supplementary file 1 [file Data_Sheet_1.PDF]

Supplementary Material

1 Supplementary Figure Captions

1.1 Supplementary Figure 1

All data used in **Figure 3A, 3B, 3C** (24h after plating). A: Measurements (size and shape) for each spheroid taken 24 hours after ChR2-HEK suspension was seeded into 96-well spheroid microplates at various densities. Group 1 (G1) denotes an initial seeding density of 2 x 10^4 cells/well, Group 2 (G2) 4 x 10^4 cells / well, Group 3 (G3) denotes an initial seeding density of 6 x 10^4 cells/well, Group 4 (G4) 8 x 10^4 cells, and Group 5 (G5) denotes an initial seeding density of 10 x 10^4 cells/well. For each seeding density, n=6. B: Corresponding brightfield and fluorescence images at 24 hours for each spheroid from which measurement analysis in (A) was performed. Scalebar: 0.5 mm.

1.2 Supplementary Figure 2

All data used in **Figure 3A, 3B, 3C** (48h after plating). A: Measurements (size and shape) for each spheroid taken 48 hours after ChR2-HEK suspension was seeded into 96-well spheroid microplates at various densities. Group 1 (G1) denotes an initial seeding density of 2 x 10^4 cells/well, Group 2 (G2) 4 x 10^4 cells / well, Group 3 (G3) denotes an initial seeding density of 6 x 10^4 cells/well, Group 4 (G4) 8 x 10^4 cells, and Group 5 (G5) denotes an initial seeding density of 10 x 10^4 cells/well. For each seeding density, n=6. B: Corresponding brightfield and fluorescence images at 48 hours for each spheroid from which measurement analysis in (A) was performed. Scalebar: 0.5 mm.

1.3 Supplementary Figure 3

All data used in **Figure 3A, 3B, 3C** (72h after plating). A: Measurements (size and shape) for each spheroid taken 72 hours after ChR2-HEK suspension was seeded into 96-well spheroid microplates at various densities. Group 1 (G1) denotes an initial seeding density of 2 x 10^4 cells/well, Group 2 (G2) 4 x 10^4 cells / well, Group 3 (G3) denotes an initial seeding density of 6 x 10^4 cells/well, Group 4 (G4) 8 x 10^4 cells, and Group 5 (G5) denotes an initial seeding density of 10 x 10^4 cells/well. For each seeding density, n=6. B: Corresponding brightfield and fluorescence images at 72 hours for each spheroid from which measurement analysis in (A) was performed. Scalebar: 0.5 mm.

1.4 Supplementary Figure 4

All data used in **Figure 3A, 3B, 3C** (96h after plating). A: Measurements (size and shape) for each spheroid taken 96 hours after ChR2-HEK suspension was seeded into 96-well spheroid microplates at various densities. Group 1 (G1) denotes an initial seeding density of 2 x 10^4 cells/well, Group 2 (G2) 4 x 10^4 cells / well, Group 3 (G3) denotes an initial seeding density of 6 x 10^4 cells/well, Group 4 (G4) 8 x 10^4 cells, and Group 5 (G5) denotes an initial seeding density of 10 x 10^4 cells/well. For each seeding density, n=6. B: Corresponding brightfield and fluorescence images at 96 hours for each spheroid from which measurement analysis in (A) was performed. Scalebar: 0.5 mm.

1.5 Supplementary Figure 5

To evaluate the viability of spheroids in culture over time, spheroids at seeding density 2 x 10^4 cells/well were cultured in a sterile propidium iodide (PI) solution of concentration 2 ug/mL in DMEM. A: WT 293T and ChR2 293T spheroids cultured without PI. B: WT 293T and ChR2 293T spheroids cultured with PI. Spheroids were imaged every 24 h under brightfield and fluorescence to reveal the PI uptake localization in the necrotic core of the spheroid. In both 293T spheroids and ChR2-293T spheroids top-down images, we observed an increase in the fraction of PI pixels to total spheroid pixels (as determined from brightfield) after day 2. These fractions were quantified over time, n = 3 spheroids per time point per group, all data points shown. Scalebar: 0.5 mm.

1.6 Supplementary Figure 6

Top-down spheroid images were run through an intensity threshold filter in order to attain the fraction of PI-stained cells. Here PI fraction is defined as the number of PI pixels with intensity above a specified threshold in the fluorescent channel over the number of pixels constituting the spheroid as determined in brightfield. Because the intensity threshold method for determining total spheroid pixels in unedited brightfield images had tendency to be inaccurate due to high frequency imperfections of the plate or other contaminants, we utilized the auto-detect ROI feature available with our imaging tool package to selectively binarize pixels that truly represented the spheroid. PI fraction was subsequently calculated from the processed images of each respective channel.

1.7 Supplementary Figure 7

All data used in **Figure 3D, 3E, 3F, 3G, 3H, 3I** (1-16 days after plating). A: Fluorescence (ChR2-eYFP) images at 8 time points over 16 days for spheroids seeded in 96-well ULA plates at (G6) 10^2 cells/well and (G7) 10^3 cells/well. B: Fluorescence (ChR2-eYFP) at 8 time points over 16 days for spheroids seeded in 384-well ULA plates at (G6) 10^2 cells/well and (G7) 10^3 cells/well. For each seeding density and each day, n=6. Size and shape measurements were calculated from the images via MATLAB. Scalebars are 0.2 mm at higher (10x) magnification and 0.5 mm at lower (4x magnification).

1.8 Supplementary Figure 8

All statistics for morphological data used in **Figure 3**. We conducted two-way ANOVAs with post-hoc Tukey or Sidak ($p < 0.05$), evaluating radius and ellipticity as a function of initial seeding density and days in culture for large spheroids seeded in 96-well plates, small spheroids seeded in 96-well plates, and small spheroids seeded in 384-well plates. Normality of the data was also tested. Here we show multiple comparisons of size and shape across seeding densities for first and last days in culture.

2 Supplementary Figures

2.1 Supplementary Figure 1

A

Group	Feature	Day 1 Measurements (μm)			
		Mean	Standard deviation	Minimum	Maximum
$2x10^4$ cells/well	Axis A	365.76	12.60	352.32	386.79
	Axis B	332.24	6.33	325.15	342.52
	Axis A/B	1.10	0.05	1.03	1.18
$4x10^4$ cells/well	Axis A	472.13	10.80	460.74	488.54
	Axis B	438.91	14.09	416.92	457.93
	Axis A/B	1.08	0.04	1.02	1.13
$6x10^4$ cells/well	Axis A	559.02	46.83	520.50	650.51
	Axis B	515.22	52.81	453.24	613.45
	Axis A/B	1.09	0.07	1.02	1.22
$8x10^4$ cells/well	Axis A	630.07	51.84	597.02	733.90
	Axis B	575.44	58.89	528.09	690.28
	Axis A/B	1.10	0.04	1.05	1.16
10^5 cells/well	Axis A	672.83	22.87	646.24	702.91
	Axis B	614.59	27.00	590.36	658.76
	Axis A/B	1.10	0.05	1.04	1.18

B

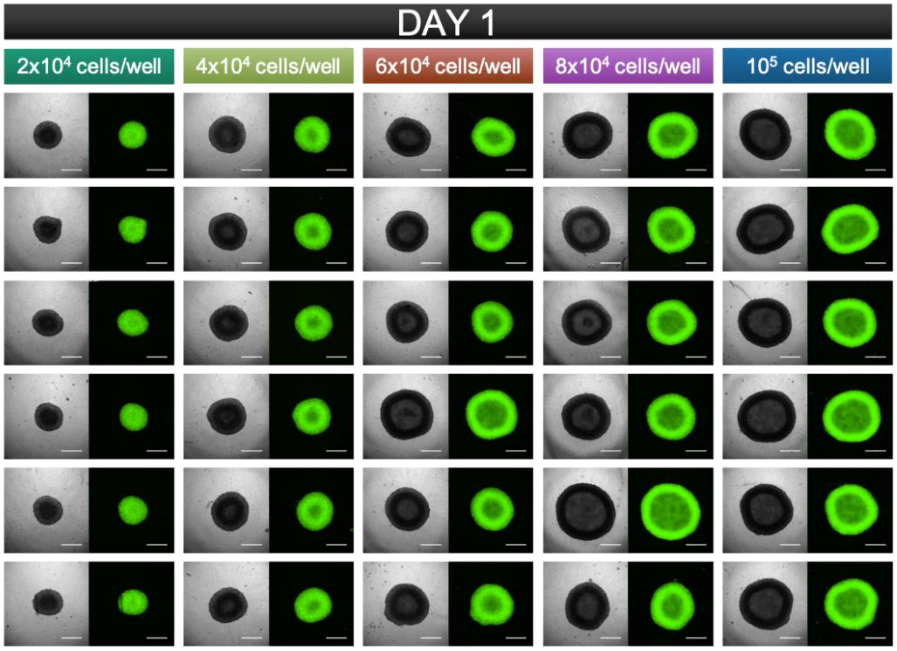

2.2 Supplementary Figure 2

A

Group	Feature	Day 2 Measurements (μm)			
		Mean	Standard deviation	Minimum	Maximum
$2x10^4$ cells/well	Axis A	362.68	8.34	353.40	372.73
	Axis B	344.04	6.02	337.07	351.71
	Axis A/B	1.06	0.03	1.01	1.08
$4x10^4$ cells/well	Axis A	463.83	8.70	452.90	473.93
	Axis B	428.05	21.60	398.85	459.48
	Axis A/B	1.09	0.06	1.02	1.19
$6x10^4$ cells/well	Axis A	519.25	30.17	489.70	574.65
	Axis B	488.00	39.03	434.12	547.85
	Axis A/B	1.07	0.05	1.04	1.16
$8x10^4$ cells/well	Axis A	570.38	46.12	533.78	651.99
	Axis B	538.90	42.82	496.06	603.96
	Axis A/B	1.06	0.02	1.03	1.08
10^5 cells/well	Axis A	596.02	16.99	578.93	618.42
	Axis B	553.75	18.34	540.33	589.45
	Axis A/B	1.08	0.02	1.05	1.11

B

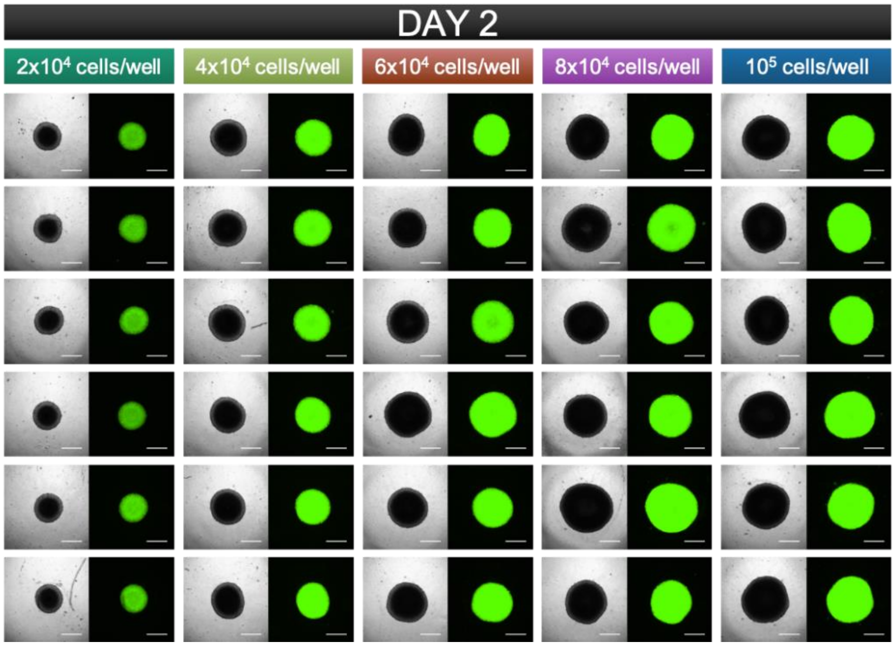

2.3 Supplementary Figure 3

A

Group	Feature	Day 3 Measurements (μm)			
		Mean	Standard deviation	Minimum	Maximum
$2x10^4$ cells/well	Axis A	398.12	12.71	388.09	418.99
	Axis B	371.84	11.34	353.81	385.59
	Axis A/B	1.07	0.06	1.01	1.18
$4x10^4$ cells/well	Axis A	480.70	22.52	457.60	521.61
	Axis B	464.15	23.75	437.18	507.26
	Axis A/B	1.04	0.02	1.01	1.06
$6x10^4$ cells/well	Axis A	544.99	55.96	494.19	649.02
	Axis B	523.66	41.87	486.90	596.69
	Axis A/B	1.04	0.03	1.01	1.09
$8x10^4$ cells/well	Axis A	609.92	66.24	537.66	714.90
	Axis B	574.78	69.61	506.30	677.96
	Axis A/B	1.06	0.04	1.02	1.12
10^5 cells/well	Axis A	583.86	28.98	551.88	621.47
	Axis B	562.30	30.61	524.10	604.42
	Axis A/B	1.04	0.02	1.01	1.05

B

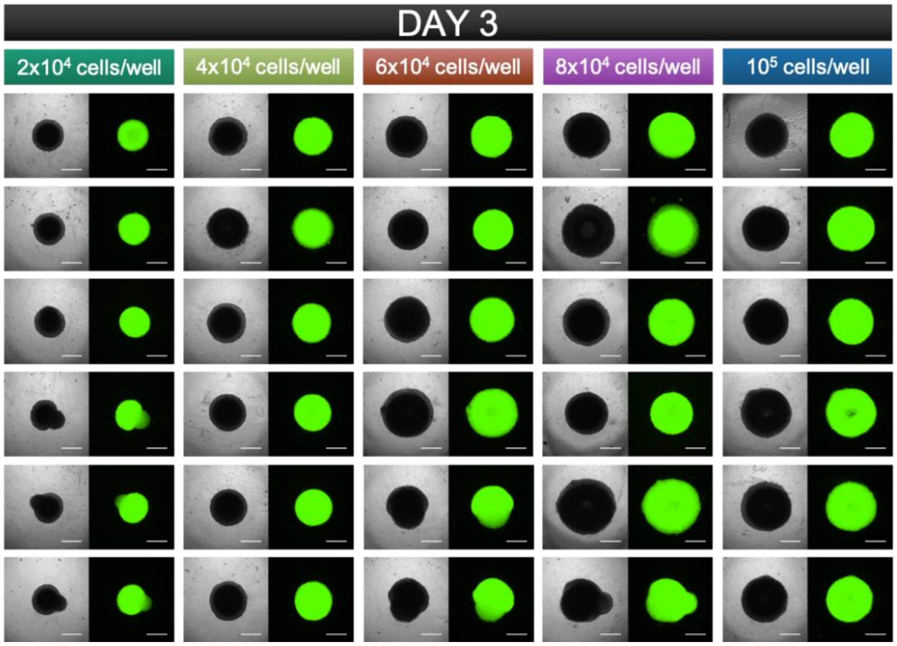

2.4 Supplementary Figure 4

A

Group	Feature	Day 4 Measurements (μm)			
		Mean	Standard deviation	Minimum	Maximum
$2x10^4$ cells/well	Axis A	450.93	38.85	409.04	504.63
	Axis B	397.22	23.11	363.83	430.88
	Axis A/B	1.14	0.12	1.01	1.31
$4x10^4$ cells/well	Axis A	525.60	32.33	495.31	566.82
	Axis B	473.79	13.21	458.12	489.00
	Axis A/B	1.11	0.09	1.02	1.23
$6x10^4$ cells/well	Axis A	626.34	68.90	528.96	686.40
	Axis B	507.77	55.06	453.76	607.19
	Axis A/B	1.25	0.21	1.03	1.49
$8x10^4$ cells/well	Axis A	691.58	99.73	538.49	814.53
	Axis B	590.14	117.38	496.35	778.04
	Axis A/B	1.19	0.21	1.02	1.56
10^5 cells/well	Axis A	697.35	75.03	588.08	788.52
	Axis B	526.15	32.12	487.56	569.66
	Axis A/B	1.34	0.21	1.11	1.62

B

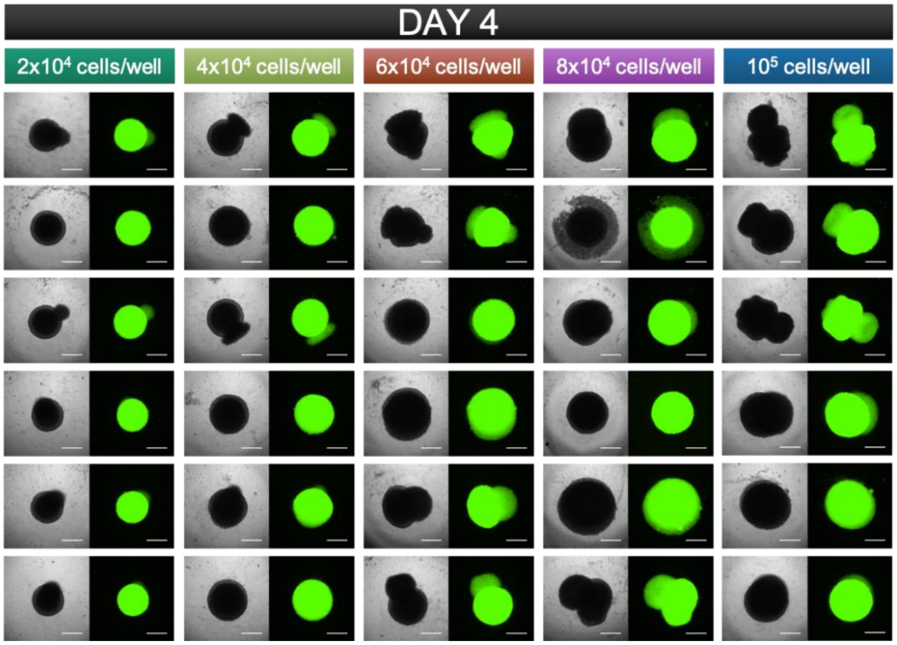

2.5 Supplementary Figure 5

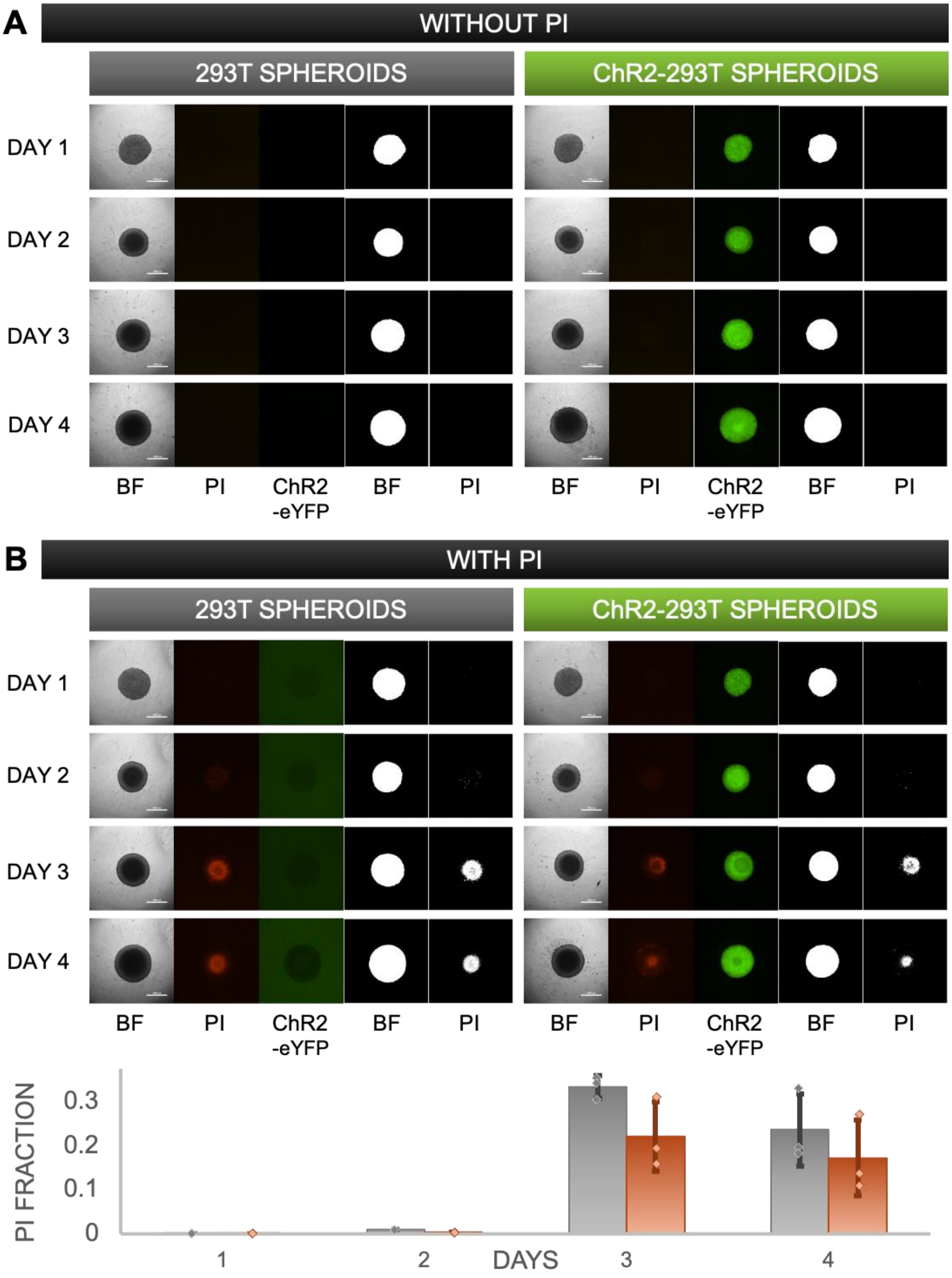

2.6 Supplementary Figure 6

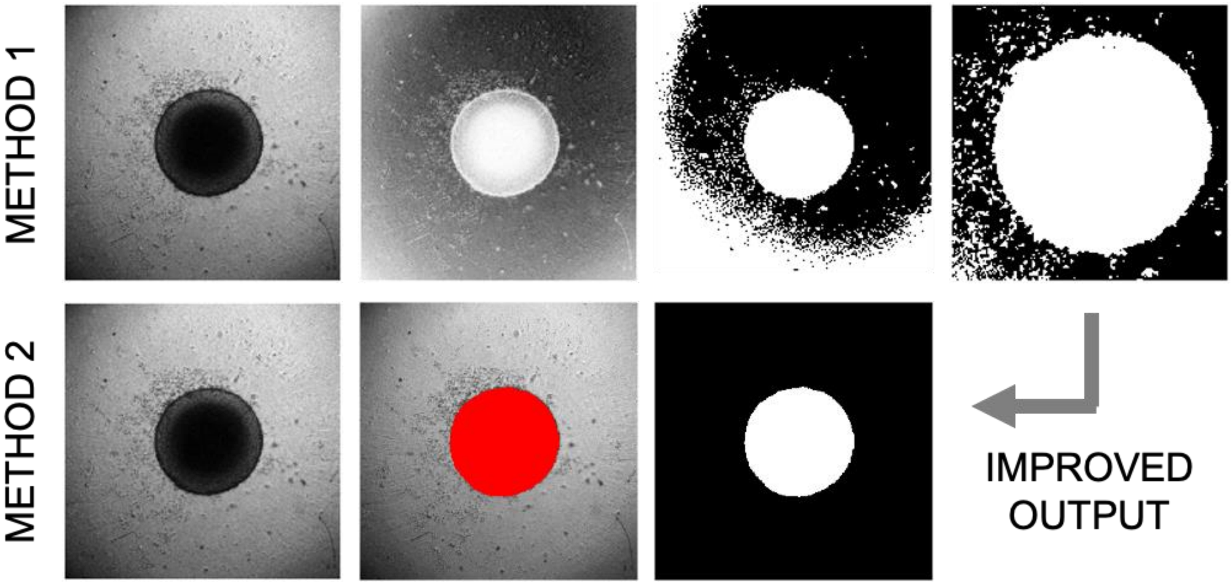

2.7 Supplementary Figure 7

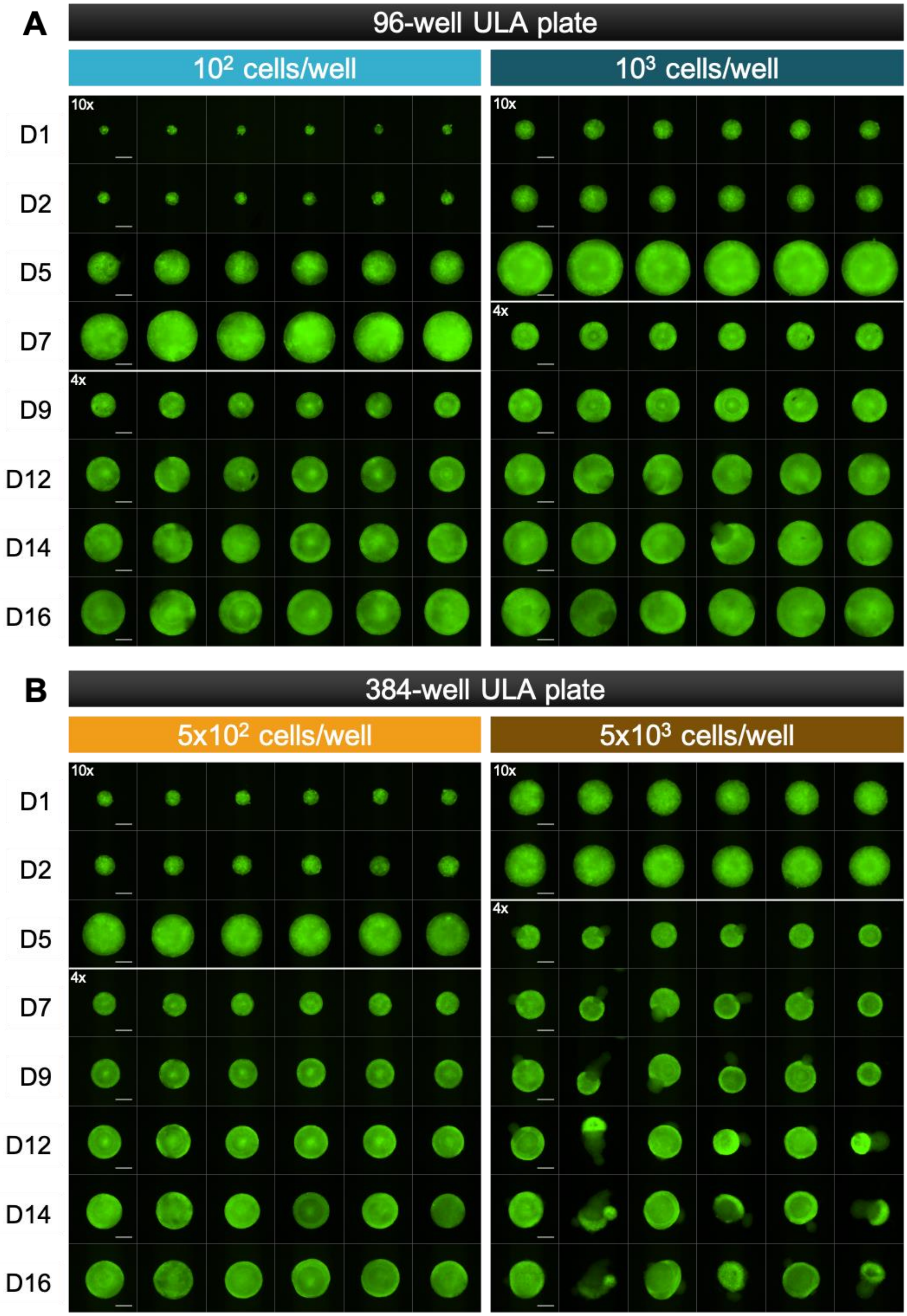

2.8 Supplementary Figure 8

LARGE SPHEROIDS (96-WELL)

RADIUS (DAY 1)

	4x10^4 cells/well	6x10^4 cells/well	8x10^4 cells/well	10^5 cells/well
2x10^4 cells/well	****	***	***	****
4x10^4 cells/well		*	**	****
6x10^4 cells/well			ns	****
8x10^4 cells/well				ns

ELLIPTICITY (DAY 1)

	4x10^4 cells/well	6x10^4 cells/well	8x10^4 cells/well	10^5 cells/well
2x10^4 cells/well	ns	ns	ns	ns
4x10^4 cells/well		ns	ns	ns
6x10^4 cells/well			ns	ns
8x10^4 cells/well				ns

RADIUS (DAY 4)

	4x10^4 cells/well	6x10^4 cells/well	8x10^4 cells/well	10^5 cells/well
2x10^4 cells/well	***	***	*	****
4x10^4 cells/well		*	ns	***
6x10^4 cells/well			ns	ns
8x10^4 cells/well				ns

ELLIPTICITY (DAY 4)

	4x10^4 cells/well	6x10^4 cells/well	8x10^4 cells/well	10^5 cells/well
2x10^4 cells/well	ns	ns	ns	ns
4x10^4 cells/well		ns	ns	ns
6x10^4 cells/well			ns	ns
8x10^4 cells/well				ns

SMALL SPHEROIDS (96-WELL)

RADIUS (DAY 1)

	10^3 cells/well
10^2 cells/well	****

ELLIPTICITY (DAY 1)

	10^3 cells/well
10^2 cells/well	**

RADIUS (DAY 16)

	10^3 cells/well
10^2 cells/well	***

ELLIPTICITY (DAY 16)

	10^3 cells/well
10^2 cells/well	ns

SMALL SPHEROIDS (384-WELL)

RADIUS (DAY 1)

	5x10^3 cells/well
5x10^2 cells/well	****

ELLIPTICITY (DAY 1)

	5x10^3 cells/well
5x10^2 cells/well	ns

RADIUS (DAY 16)

	5x10^3 cells/well
5x10^2 cells/well	ns

ELLIPTICITY (DAY 16)

	5x10^3 cells/well
5x10^2 cells/well	ns
